# Diagnostic mRNA splicing assay for variants in *BRCA1* and *BRCA2* identified two novel pathogenic splicing aberrations

**DOI:** 10.1186/s13053-019-0113-9

**Published:** 2019-05-22

**Authors:** Teresia Wangensteen, Caroline Nangota Felde, Deeqa Ahmed, Lovise Mæhle, Sarah Louise Ariansen

**Affiliations:** Department of Medical Genetics, Oslo University Hospital, Oslo, Norway

**Keywords:** *BRCA1*, *BRCA2*, mRNA, Splicing, RT-PCR, Variant of unknown significance, Variant classification

## Abstract

**Background:**

Pathogenic variants in *BRCA1* and *BRCA2* cause hereditary breast and ovarian cancer. Screening of these genes has become easily accessible in diagnostic laboratories. Sequencing and copy number analyses are used to detect pathogenic variants, but also lead to identification of variants of unknown clinical significance (VUS). If the effect of a VUS can be clarified, it has direct consequence for the clinical management of the patient and family members. A splicing assay is one of several tools that might help in the classification of VUS. We therefore established mRNA analyses for *BRCA1* and *BRCA2* in the diagnostic laboratory in 2015. We hereby report the results of mRNA analysis variants in *BRCA1* and *BRCA2* after three years.

**Methods:**

Variants predicted to alter splicing and variants within the canonical splice sites were selected for splicing analyses. Splicing assays were performed by reverse transcription-PCR of patient RNA. A biallalic expression analysis was carried out whenever possible.

**Results:**

Twenty-five variants in *BRCA1* and *BRCA2* were analyzed by splicing assays; nine showed altered transcripts and 16 showed normal splicing patterns. The two novel pathogenic variants in *BRCA1* c.4484 + 3 A > C and c.5407–10G > A were characterized.

**Conclusions:**

We conclude that mRNA analyses are useful in characterization of variants that may affect splicing. The results can guide classification of variants from unknown clinical significance to pathogenic or benign in a diagnostic laboratory, and thus be of direct clinical importance.

## Background

Pathogenic variants in *BRCA1* and *BRCA2* cause hereditary breast and ovarian cancer. Since the discovery of the genes more than 20 years ago [1–3], clinical testing has gradually become more available and comprehensive. As the methods have developed, resulting in cheaper and faster analyses, more patients are given the opportunity to have a genetic test and the tests have expanded from a few founder mutations to full gene testing of *BRCA1* and *BRCA2*, and more recently additional genes.

Full sequencing and copy number analyses of *BRCA1* and *BRCA2* have the obvious advantage of finding more disease causing variants compared to a limited analysis. A disadvantage is the identification of variants of unknown clinical significance (VUS), and variant interpretation has become a major challenge.

It is of great importance to investigate, if possible, if a variant is pathogenic or not, as it may affect the clinical management of the patient and relatives, including disease screening, prevention and treatment. Multifactorial models have been developed to help classify variants. The international consortium ENIGMA (Evidence-Based Network for the Interpretation of Germline Mutant Alleles) was initiated in 2009 [4]. A main goal was to assess the association of genetic variants with cancer predisposition through collaboration between researchers and clinicians, collecting and combining information on genetic variants in the breast cancer genes. The multifactorial method integrates data from several different independent sources such as family history, cosegregation with disease in families, and co-occurrence of a variant in trans with a pathogenic mutation. This is a favourable approach when there is enough available information on a specific variant. However, many variants are rare, and gathering enough information to reach a conclusion regarding pathogenicity in a multifactorial analysis is not always possible. Suitable functional analyses may therefore be of great value.

For exonic variants there are several ways in which a variant can alter the protein or its function, including missense, nonsense and frameshift. In addition to this, any variant, both exonic and intronic, can potentially affect splicing [5]. There are still limitations in knowledge and tools to identify all variants that alter splicing. However, we do have bioinformatic tools to predict splicing effects of variants in or near the consensus splice site with reasonable sensitivity and specificity [6]. These bioinformatic prediction tools are useful to select variants for functional splicing analysis, which can then confirm or disprove the predicted effect.

A variant within a consensus splice site will usually be reported as a variant of unknown significance unless additional information is available, even if there is no prediction of altered splicing. In this setting, a splicing assay can be valuable in reclassification from a VUS to a benign variant. Variants within the canonical splice site will usually be classified as likely pathogenic unless other information is available. Most canonical splice site variants are pathogenic, but there are known exceptions. Some variants within the canonical splice sites lead to naturally occurring in-frame isoforms that may rescue gene functionality. For *BRCA1* and *BRCA2* such variants are listed in the ENIGMA *BRCA1/2* Gene Variant Classification Criteria (Table 6 in version 2.5.12017). So even in the case of a canonical splice site variant, a functional study to confirm -or possibly disprove- the effect on splicing is of great value.

Our laboratory established a diagnostic mRNA splicing assay for *BRCA1* and *BRCA2* in 2015, based on the recommendations published by ENIGMA [7]. Variants predicted to lead to loss of a splice site, introduction of a new splice site, or activation of a cryptic splice site and variants within the consensus splice site were selected for mRNA splicing analysis. Here we report the results after three years of diagnostic functional splicing analysis for *BRCA1* and *BRCA2*.

## Methods

### Selection criteria for RNA analysis

Variants were selected for RNA analysis based on the results of sequencing of *BRCA1* and *BRCA2* in patients undergoing diagnostic or predictive genetic testing for hereditary breast and ovarian cancer. Variants within a consensus splice site were selected for RNA analysis, regardless of splicing prediction as recommended by ENIGMA [8]. A consensus splice site was defined as the last three nucleotides of the exon and the first six of the intron (donor splice site) and last twenty nucleotides of an intron and the two first of the next exon (acceptor splice site) [9].

Identified variants that were predicted to disrupt or create a splice site were also included regardless of their localization within or outside splice sites. The Alamut software was used for splicing predictions. The four tools SpliceSiteFinder-like, MaxEntScan, NNSPLICE and GeneSplicer, were used, on the basis of a resource analysis by National Genetics Reference Laboratory in Manchester “Splice Site Tools, A Comparative Analysis Report by Hellen B (http://www.ngrl.org.uk/Manchester/)”. We considered a reduction in the splice site score by 10–20% by at least three of the tools as significant. The score of the normal splice site was taken into account. The prediction of a new splice site was considered significant if at least three of the tools recognized the putative new splice site. Again, the score, and the distance to the normal splice site was considered. When in doubt, we had a liberal approach in choosing variants for RNA analysis. We also included some variants where the literature showed divergent or inconclusive results, even though the variant did not meet our criteria for splicing assay.

As we were establishing a new method in our laboratory, we decided to include variants that had been analyzed by splicing assays by others for the purpose of validation.

### Nomenclature

The DNA and mRNA variant numbering is based on the cDNA sequence for *BRCA1* (GeneBank: NM_007294.3) and *BRCA2* (GeneBank: NM_000059.3). The *BRCA1* and *BRCA2* nomenclature follows the recommendations from the Human Genome Variation Society (HGVS), where A in the translation initiation codon ATG is base c.1 (http://varnomen.hgvs.org/). Exons are numbered systematically (1…n) in *BRCA1* and *BRCA2*, which may differ from previous publications where exon numbering of *BRCA1* is based on historical numbering of exons (1,2,3,5…n).

### Blood samples, RNA extraction and reverse transcriptase PCR

Blood from patients undergoing genetic testing at Oslo University Hospital, and control blood samples from voluntary blood bank donors, was drawn and stored in PAXgene Blood RNA tubes according to manufacturer’s recommendations. RNA was extracted using the PAXgene Blood RNA kit (both from Preanalytix, Qiagen, Hombreachtikon, Switzerland), according to the recommended protocols (PreAnalytiX) including DNAse treatment. cDNA was generated using the SuperScript Vilo cDNA Synthesis Kit (Invitrogen, Carlsbad, California, USA), using 150 ng input RNA.

### Transcript analysis

Transcript analysis was carried out by recommendations given by ENIGMA [7]. PCR for fragment analysis was performed using AmpliTaq Gold 360 DNA Polymerase (Applied Biosystems, Foster City, California, USA). Reverse primers were tagged with FAM at the 5′ end for detection by Capillary electrophoresis (CE). The PCR fragments were separated by CE on ABI 3730xl DNA analyzer and using GeneScan™ 1200 LIZ® dye Size Standard (both from Applied Biosystems). CE analysis was performed with GeneMarker software (SoftGenetics, Pennsylvania, USA).

All patient samples were amplified in duplicates. Two to three controls from individuals not carrying the variant were always amplified in parallel with patient samples. In order to avoid allele drop out due to failure of primers to anneal, all patients were checked for single nucleotide polymorphisms (SNPs) in the primer annealing sequence. Sequencing was carried out on ABI 3730xl DNA analyzer (Applied Biosystems) using BigDye Terminator v3.1 Cycle Sequencing Kit (Applied Biosystems).

For variants with that affected splicing, we assessed if the variant induced partial or complete splicing aberration, as recommended by ENIGMA [9]. This was achieved by specifically amplifying the wild-type transcript including a heterozygous exonic variant, whenever this was possible. In addition, if possible, a region including a heterozygous exonic variant was amplified in order to determine expression of both alleles in samples with no effect on splicing.

Based on the results of the analyses, each variant was given a splice class as defined by Houdayer et al. [6]; Class 3S: Severe impact on splicing/the mutant allele does not produce the wild type transcript, complete effect, 2S: impact on alternative splicing/leaky splice site mutation, partial effect, and Class 1S: no effect on splicing with identification of the two alleles using informative SNPs.

## Results

Out of the 25 variants analyzed in the splicing assay, nine showed altered splicing (Table 1). All nine of the aberrant transcripts had altered reading frames, eventually leading to premature stop codons. Five of these had been assessed in different functional splicing assays before, and our results were consistent with those of other studies. Four of the variants have not been characterized by functional studies before, as far as we know. Two of these showed complete pathogenic splicing aberrations. Unfortunately, for the other two novel variants we were not able to assess whether the altered splicing was partial or complete. Sixteen variants had normal splicing pattern (Table 2). We were able to demonstrate biallelic expression for 12 of these sixteen variants.

**Table 1:** Variants with altered transcripts

Gene	HGVS	Prediction/indication	Result mRNA	Splice class	Consequence	Previous publication and conclusion
*BRCA1*	c.4484G > A	Reduced donor site	r.4358_4484del, p.(Ala1453Glyfs*10)	NA	Exon 13 skipped out of frame	Houdayer 2012: severe impact on splicing (RT-PCR) (“Exon 14 skipped”)
*BRCA1*	c.4484 + 3A > C	Reduced donor site	r.4358_4484del, p.(Ala1453Glyfs*10)	3S	Exon 13 skipped, out of frame, complete	
*BRCA1*	c.4675G > A	Loss of donor site	r.4665_4675del, p.Gln1556Glyfs*14	3S	Loss of last 11 bp in exon 14, out of frame, complete	Wappenschmidt 2012: severe impact on splicing (RT-PCR) (“D11nt 3′ of exon 15”)
*BRCA1*	c.5332 + 4A > G	Reduces donor site	r.5278_5332del, p.(Phe1761Asnfs*14)	NA	Exon 20 skipped, out of frame	
*BRCA1*	c.5407–10G > A	Loss of acceptor + activation of cryptic	r.5406_5407ins5407-8_5407–1, p.(Val1804Serfs*33)	3S	Retention of 8 bp of intron 21, out of frame, complete	
*BRCA2*	c.631 + 4A > G	Reduced donor site	r. 517_631del, p.(Gly173Serfs*19)	3S	Exon 7 skipped, out of frame, complete	Steffensen 2010: disease causing (minigene + RT-PCR)
*BRCA2*	c.7992 T > A	Slightly reduced acceptor site	r.7977_8331del p.(Tyr2660Phefs*43)	2S	Exon 18 skipped, out of frame, partial	Fackenthal 2016: minor alternate transcript (∆18) (RT-PCR)
*BRCA2*	c.8331 + 2 T > C	Reduced donor site	r.7977_8331del p.(Tyr2660Phefs*43)	NA	Exon 18 skipped, out of frame, complete	Fraile-Bethencourt 2017: pathogenic (minigene)
*BRCA2*	c.8754 + 5G > C	Reduced donor site + activation of cryptic	r.8754_8755ins8754 + 1_8754 + 46, p.Gly2919Valfs*4	NA	Retention of 46 bp of intron 21, out of frame	

Splice class as defined by Houdayer 2012; 3S: Severe impact on splicing/the mutant allele does not produce the wildtype transcript, complete effect, 2S: impact on alternative splicing/leaky splice site mutation, partial effect, *NA* not analyzed

**Table 2:** Variants with normal transcripts

Gene	HGVS	Prediction/indiation	Splice classs	Previous splicing assay
*BRCA1*	c.80 + 16A > T	Possibly new cryptic donor site	NA	
*BRCA1*	c.81-13C > G	Reduced acceptor site	1S	Houdayer 2012
*BRCA1*	c.81-14C > G	Reduced acceptor site	NA	
*BRCA1*	c.302-15C > G	In consensus splice site	1S	Steffensen 2014
*BRCA1*	c.441G > C p.(Leu147Phe)	In consensus splice site	1S	
*BRCA1*	c.594-20A > G	In consensus splice site	1S	
*BRCA1*	c.4676-8C > G	Reduced acceptor site	1S	
*BRCA1*	c.4987-4 T > G	In consensus splice site	1S	
*BRCA1*	c.5333-17C > A	In consensus splice site	NA	
*BRCA2*	c.521G > A p.(Arg174His)	Diverging splicing results in publications	1S	Houdayer 2012, Di Giacomo 2013
*BRCA2*	c.682-12_682-11delTA	In consensus splice site	1S	Spearman 2008
*BRCA2*	c.7006C > T p.(Arg2336Cys)	In consensus splice site	1S	
*BRCA2*	c.7436-4A > G	In consensus splice site	1S	
*BRCA2*	c.9234C > T p.(Val3078=)	Partial exon 24 skipping published	1S	Sanz 2010
*BRCA2*	c.9257G > C p.(Gly3086Ala)	In consensus splice site	1S	
*BRCA2*	c.9502-12 T > G	In consensus splice site	NA	Houdayer 2012, Joosse 2012, Acedo 2015

Splice class as defined by Houdayer 2012; Class 1S: no effect on splicing with identification of the two alleles using informative single nucleotide polymorphisms (SNPs), NA = not analyzed

### Variants with altered transcripts

**Fig. 1:**
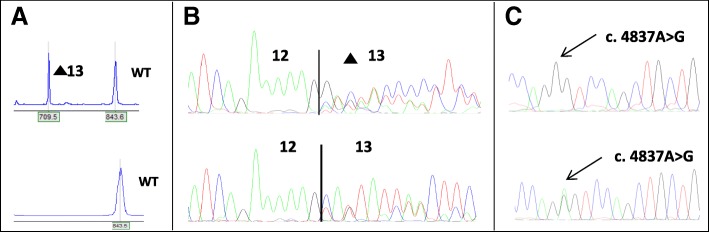
Results of the analyses of *BRCA1* c.4484 + 3A > C. **a**) Result of fragment analyses of RT-PCR of patient and control. Analysis of RT-PCR products of the patient and control sample showed one fragment with the expected product size in both samples, in addition to one shorter fragment which is only present in the patient sample (top). **b**) Result of sequencing of fragments from RT-PCR in patient and control. Electropherogram of the sequence of the RT-PCR product from the patient showing a transcript lacking exon 13, in addition to the full length transcript (top). Both samples also show the known alternative transcript, r.4358_4360del, lacking the first three bases of exon 13 (called Δ14 in ENIGMA report by Colombo et al. 2014). **c**) Results of investigation of biallelic expression. Investigation of biallelic expression using primers specific for amplification of wild type transcript shows monoallelic expression as a pure G appears at position c.4837 in the cDNA (top), compared to the heterozygous pattern in the patient DNA (below). The result indicates that the mutated allele does not produce WT-transcript

**Fig. 2:**
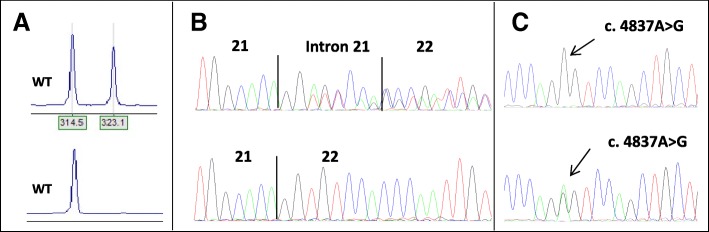
Results of the analyses of *BRCA1* c.5407–10G > A. **a**) Result of fragment analyses of RT-PCR of patient and control Analysis of RT-PCR products of the patient and control sample showed one fragment with the expected product size in both samples, in addition to one longer fragment which is only present in the patient sample (top). **b**) Result of sequencing of fragments from RT-PCR in patient and control. Electropherogram of the sequence of the RT-PCR product from the patient (top) showing a transcript with retention of eight bases of intron 21, in addition to the full length transcript. **c**) Results of investigation of biallelic expression. Investigation of biallelic expression using primers specific for amplification of wild type transcript shows monoallelic expression as a pure G appears at position c.4837 in the cDNA (top), compared to the heterozygous pattern in the patient DNA (below). The result indicates that the mutated allele does not produce WT-transcript

Two novel pathogen splicing variants in *BRCA1* were identified; c.4484 + 3 A > C and c.5407–10G > A. The variant *BRCA1* c.4484 + 3A > C led to complete skipping of exon 14, which results in a disruption of the open reading frame (Fig. 1). The variant *BRCA1* c.5407–10G > A showed retention of 8 bases of intron 22 i the mutant transcript (Fig. 2). There was no normal transcript produced from the variant alleles.

Two additional novel variants leading to abnormal splicing were identified, *BRCA1* c.5332 + 4A > G and *BRCA2* c.8754 + 5G > C, unfortunately we were not able to show if the aberration was complete or partial in these cases and could thus not draw any conclusion about pathogenicity. *BRCA1* c.5332 + 4A > G led to skipping of exon 20, which disrupts the open reading frame. *BRCA2* c.8754 + 5G > C led to retention of 46 bases of the 5′-end of intron 21 in the mutant transcript.

Five of the variants with altered splicing had been published before. The detected transcripts and references to the literature are listed in Table 1. Four were pathogenic due to complete aberrations of the reading frame; *BRCA1* c.4484G > A led to skipping of exon 13. *BRCA1* c.4675G > A led to a complete loss of the last 11bases of exon 14. *BRCA2* c.631 + 4A > G led to complete skipping of exon 7. *BRCA2* c.8331 + 2 T > C led to a complete skipping of exon 18, whereas *BRCA2* c.7992 T > A led to a partial skipping of exon 18, which is a naturally occurring minor alternative transcript. *BRCA2* c.7992 T > A increased the amount of this alternative transcript.

### Variants with normal transcripts

Sixteen variants showed normal splicing (Table 2). Twelve of them were demonstrated to have normal transcripts produced by both alleles. Of these, seven were intronic variants and one was a synonymous exonic variant. These eight variants could be classified as benign on the basis of the splicing results. Four of these variants; *BRCA1* c.594-20A > G, c.4676-8C > G and c.4987-4 T > G and *BRCA2* c.7436-4A > G had not been analyzed by a splicing assay before as far as we know.

## Discussion

We report the results of a splicing assay for a number of variants, some with aberrations and some with normal results. In both cases, the results are of significant clinical utility.

All the detected abnormal transcripts resulted in premature stop codons. Transcripts containing premature termination codons are degraded by nonsense-mediated mRNA decay (NMD), unless they are localized less than 50 nucleotides upstream of the position of the terminal intron [10, 11]. We concluded that the variants that gave rise to only transcripts with premature stop codons are pathogenic as they are all appear in parts of the gene where we expect them to be degraded by NMD.

We characterized two novel pathogenic splicing variants, *BRCA1* c.5407–10G > A and c.4484 + 3A > C. *BRCA1* c.5407–10G > A had been reported as VUS in the ClinVar database (a database on the relationships between human variations and phenotypes) and listed once in the genome Aggregation Database (gnomAD) (> 246,000 alleles). *BRCA1* c.4484 + 3A > C had not been reported before to our knowledge, neither in publications nor in databases. We classified these two variants as pathogenic as both led to complete splicing aberrations which altered the reading frame leading to premature stop codon; *BRCA1* c.5407–10G > A through retention of eight intronic bases, and *BRCA1* c.4484 + 3A > C thorough skipping of exon 13 (the same transcript as for *BRCA1* c.4484G > A). In these two cases the mRNA results directly affected the clinical management of the patient. In addition, their relatives were given the opportunity to undergo predictive genetics testing, giving carriers the possibility to opt for disease screening and/or prevention.

For the other two novel variants with abnormal splicing, we were unfortunately not able to draw any conclusions about pathogenicity. *BRCA1* c.5332 + 4A > G had been reported in ClinVar, three times as a variant of unknown significance and one registration as likely pathogen by CIMBA, but without provided evidence for the conclusion. *BRCA1* c.5332 + 4A > G was listed four times in gnomAD (> 246,000 alleles). *BRCA2* c.8754 + 5G > C led to retention of 46 bases of the 5′-end of intron 21 in the mutant transcript. This variant has not been reported before to our knowledge, neither in publications, nor in databases, but the same transcript has previously been found for another pathogenic variant in the close vicinity, namely *BRCA2* c.8754 + 3 [12].

Five of the variants with aberrant splicing had been published before. The methods used for splicing assay were slightly different, but we detected the same transcripts and came to the same conclusions regarding pathogenicity for these variants. The two known pathogenic *BRCA1* variants, c.4484G > A and c.4675G > A, had both been published with functional studies in patient RNA in 2012 [6, 13]. They have since been reported by different laboratories in ClinVar and in different populations. *BRCA1* c.4484G > A leading to skipping of exon 13 (previously known as exon 14) has been reported in patients in France [14], Australia [15] and Brasil [16]. *BRCA1* c.4675G > A leading to loss of the last 11bases of exon 14 (previously known as exon 15) has been reported in patients in Germany [13] and Lithuania [17]. The variant *BRCA2* c.631 + 4A > G was found in a Danish breast and ovarian cancer family and analyzed with a combination of minigene and RT-PCR with the conclusion of a disease causing mutation [18]. The variant *BRCA2* c.8331 + 2 T > C has been reported several times in ClinVar and is assumed to be pathogenic because of the localization in the canonical donor splice site. However, it was only in 2017, that the result of a functional assay was published, a mingene assay that demonstrated 87% exon 18 skipping [19]. We confirmed a complete pathogenic exon 18 skipping in a patient sample.

*BRCA2* c.7992 T > A led to a partial skipping of exon 18, which is a naturally occurring minor alternative transcript [20]. *BRCA2* c.7992 T>A increased the amount of the alternative transcript lacking exon 18. This variant remains a variant of unknown significance, consistent with the conclusion drawn by Fraile-Bethencourt et al. [19]. They quantified the ratio between the transcripts produced by this variant in a minigene assay and found 69% of the normal transcript and 31% of the transcript lacking exon 18.

Sixteen variants showed normal splicing. Twelve of them were demonstrated to have normal transcripts produced by both alleles. Of these, seven were intronic variants and one was a synonymous exonic variant. We classified these eight variants as benign based on the results of the splicing assay. To our knowledge, we are the first to report the results of a splicing assay for four of these, including *BRCA1* c.594-20A > G, c.4676-8C > G and c.4987-4 T > G and *BRCA2* c.7436-4A > G. These variants are rare (reported in gnomAD once, eighteen, two and four times, respectively).

Four of the variants with normal splicing pattern were exonic variants leading to amino acid substitutions, which have to be assessed separately regarding a potential effect on protein function, but we can at least rule out major splicing aberrations. Three of these exonic variants (*BRCA1* c.441G > C p.(Leu147Phe) and *BRCA2* c.7006C > T p.(Arg2336Cys) and c.9257G > C p.(Gly3086Ala)) had not been analyzed in a splicing assay before, as far as we know.

Three *BRCA1* variants c.81-13C > G, c.81-14C > G and c.4676-8C > G were predicted to attenuate the acceptor splice site. For two of these, c.81-13C > G and c.4676-8C > G, we and others, demonstrated normal splicing. For *BRCA1* c.81-14C > G, we detected only normal transcripts, but were unfortunately not able to demonstrate biallelic expression and could therefore not draw a firm conclusion. *BRCA1* c.81–13 lies in the polypyrimidine tract. Even though the substitution of a C with a G interferes with the polypyrimidine stretch, there are still 16 pyrimidines in a row, so we assume that the substitution does not affect the qualities of the tract. The same argument would apply for *BRCA1* c.81-14C > G. For the variant *BRCA1* c.4676-8C > G, the pyrimidne tract is shorter, but closer to the intron-exon boundery, which could be an explenation for preserved normal splicing [21].

The fact that several of the variants, both the pathogenic and the normal ones, are very rare, illustrate the need for functional studies for variant classification in these cases.

When it comes to selecting variants for splicing assays, ideally one should analyze all variants, as any variant could potentially affect splicing [5]. There is no efficient way to do this yet. Sensitivity of splicing prediction tools is highest in the vicinity of consensus splice sites, whereas predictions of variant effect on splicing enhancers, silencers and branchpoint have poor specificity [6]. Bioinformatic predictions must be confirmed by functional mRNA analysis.

Overall, there was a good concordance between splicing predictions and results of mRNA analyses. All variants that altered transcription were predicted to do so. However, in line with others, we demonstrated that even if the bioinformatic tools are successful in predicting reduction in a splice site, they may not be able to discriminate between activation of a new site versus exon skipping [8]. For the variants that maintained a normal splicing pattern, a few were predicted to alter splicing. These were located in the polypyrimidine tract. It is known that the polypyrimidine tract is quite variable from gene to gene and intron to intron. The function of the polypyrimidine tract is dependent in a number of factors, including total length of the tract, number of consecutive pyrimidines, distance to the intron-exon boundary, distance to the branch point and strength of the branch point [21, 22]. It seems that the variation in all these factors might make it difficult to predict whether a single change in the polypyrimidine tract results in altered splicing or not, by the bioinformatic tools, giving us another example of the benefit of splicing assays.

Minigene assays are valuable in assessing splicing effects of a variant, especially if there is no available patient mRNA [19, 23]. An advantage with this method is the investigation of the expression of one allele at a time. However, as the transcription takes place in a different environment than a human cell, and discrepancies in results from minigenes and patient samples have been reported, confirmation in patient RNA is recommended if possible. We confirmed the result of minigene reported by others in patient RNA for a couple of the variants. A disadvantage with the patient RNA method is the need for a normal variant in the patient sample, to be able to discriminate between the alleles in the distinction between partial or complete splicing aberration, and the assessment of biallelic expression in normal transcripts.

As the splicing process could be differently regulated in different tissues, a sample from breast or ovarian tissue would be ideal for the study of splicing variants in the breast cancer genes. This is usually not available in the clinical setting. However, ENIGMA concluded that *BRCA1* alternative splicing is similar in blood and breast, supporting the clinical relevance of blood based in vitro splicing assays [24].

The methods used here were shown to work well and help in the functional assessment of several variants. However, there are numbers of potential splicing variants that we are missing because of lack of efficient ways of identifying these, such as variants affecting splicing enhancers and silencers. These and other regulatory variants will probably be better characterized in the near future.

## Conclusions

We conclude that mRNA analyses are beneficial in characterization of variants that may affect splicing. The results can guide classification of variants from unknown clinical significance to pathogenic or benign, and thus be of great importance to patients and relatives, enabling female carriers to opt for disease screening and/or prevention. We characterized two novel pathogenic splicing variants. Development of knowledge and methods in the field will lead to identification and characterization of more splicing variants in the future.

## Abbreviations

ENIGMA: Evidence-Based Network for the Interpretation of Germline Mutant Alleles
gnomAD: genome Aggregation Database
NMD: Nonsense-mediated mRNA decay
RT-PCR: Reverse transcription polymerase chain reaction
SNP: Single nucleotide polymorphisms
VUS: Variant of unknown clinical significance
WT: Wild type
BRCA1/2: Breast cancer gene 1/2

## References

[1] Hall JM, Lee MK, Newman B, Morrow JE, Anderson LA, Huey B (1990). Linkage of early-onset familial breast cancer to chromosome 17q21. Science..

[2] Miki Y, Swensen J, Shattuck-Eidens D, Futreal PA, Harshman K, Tavtigian S (1994). A strong candidate for the breast and ovarian cancer susceptibility gene BRCA1. Science..

[3] Wooster R, Bignell G, Lancaster J, Swift S, Seal S, Mangion J (1995). Identification of the breast cancer susceptibility gene BRCA2. Nature..

[4] Spurdle AB, Healey S, Devereau A, Hogervorst FB, Monteiro AN, Nathanson KL (2012). ENIGMA--evidence-based network for the interpretation of germline mutant alleles: an international initiative to evaluate risk and clinical significance associated with sequence variation in BRCA1 and BRCA2 genes. Hum Mutat.

[5] Sanz DJ, Acedo A, Infante M, Duran M, Perez-Cabornero L, Esteban-Cardenosa E (2010). A high proportion of DNA variants of BRCA1 and BRCA2 is associated with aberrant splicing in breast/ovarian cancer patients. Clin Cancer Res.

[6] Houdayer C, Caux-Moncoutier V, Krieger S, Barrois M, Bonnet F, Bourdon V (2012). Guidelines for splicing analysis in molecular diagnosis derived from a set of 327 combined in silico/in vitro studies on BRCA1 and BRCA2 variants. Hum Mutat.

[7] Whiley PJ, de la Hoya M, Thomassen M, Becker A, Brandao R, Pedersen IS (2014). Comparison of mRNA splicing assay protocols across multiple laboratories: recommendations for best practice in standardized clinical testing. Clin Chem.

[8] Thomassen M, Blanco A, Montagna M, Hansen TV, Pedersen IS, Gutierrez-Enriquez S (2012). Characterization of BRCA1 and BRCA2 splicing variants: a collaborative report by ENIGMA consortium members. Breast Cancer Res Treat.

[9] Walker LC, Whiley PJ, Houdayer C, Hansen TV, Vega A, Santamarina M (2013). Evaluation of a 5-tier scheme proposed for classification of sequence variants using bioinformatic and splicing assay data: inter-reviewer variability and promotion of minimum reporting guidelines. Hum Mutat.

[10] Perrin-Vidoz L, Sinilnikova OM, Stoppa-Lyonnet D, Lenoir GM, Mazoyer S (2002). The nonsense-mediated mRNA decay pathway triggers degradation of most BRCA1 mRNAs bearing premature termination codons. Hum Mol Genet.

[11] Faustino NA, Cooper TA (2003). Pre-mRNA splicing and human disease. Genes Dev.

[12] Colombo M, De Vecchi G, Caleca L, Foglia C, Ripamonti CB, Ficarazzi F (2013). Comparative in vitro and in silico analyses of variants in splicing regions of BRCA1 and BRCA2 genes and characterization of novel pathogenic mutations. PLoS One.

[13] Wappenschmidt B, Becker AA, Hauke J, Weber U, Engert S, Kohler J (2012). Analysis of 30 putative BRCA1 splicing mutations in hereditary breast and ovarian cancer families identifies exonic splice site mutations that escape in silico prediction. PLoS One.

[14] Lecarpentier J, Nogues C, Mouret-Fourme E, Gauthier-Villars M, Lasset C, Fricker JP (2012). Variation in breast cancer risk associated with factors related to pregnancies according to truncating mutation location, in the French national BRCA1 and BRCA2 mutations carrier cohort (GENEPSO). Breast Cancer Res.

[15] Alsop K, Fereday S, Meldrum C, deFazio A, Emmanuel C, George J (2012). BRCA mutation frequency and patterns of treatment response in BRCA mutation-positive women with ovarian cancer: a report from the Australian ovarian Cancer study group. J Clin Oncol.

[16] Fernandes GC, Michelli RA, Galvao HC, Paula AE, Pereira R, Andrade CE (2016). Prevalence of BRCA1/BRCA2 mutations in a Brazilian population sample at-risk for hereditary breast cancer and characterization of its genetic ancestry. Oncotarget..

[17] Janavicius R, Rudaitis V, Mickys U, Elsakov P, Griskevicius L (2014). Comprehensive BRCA1 and BRCA2 mutational profile in Lithuania. Cancer Genet.

[18] Steffensen AY, Jonson L, Ejlertsen B, Gerdes AM, Nielsen FC, Hansen TV (2010). Identification of a Danish breast/ovarian cancer family double heterozygote for BRCA1 and BRCA2 mutations. Familial Cancer.

[19] Fraile-Bethencourt E, Diez-Gomez B, Velasquez-Zapata V, Acedo A, Sanz DJ, Velasco EA (2017). Functional classification of DNA variants by hybrid minigenes: identification of 30 spliceogenic variants of BRCA2 exons 17 and 18. PLoS Genet.

[20] Fackenthal JD, Yoshimatsu T, Zhang B, de Garibay GR, Colombo M, De Vecchi G (2016). Naturally occurring BRCA2 alternative mRNA splicing events in clinically relevant samples. J Med Genet.

[21] Coolidge CJ, Seely RJ, Patton JG (1997). Functional analysis of the polypyrimidine tract in pre-mRNA splicing. Nucleic Acids Res.

[22] Roscigno RF, Weiner M, Garcia-Blanco MA (1993). A mutational analysis of the polypyrimidine tract of introns. Effects of sequence differences in pyrimidine tracts on splicing. J Biol Chem.

[23] Steffensen AY, Dandanell M, Jonson L, Ejlertsen B, Gerdes AM, Nielsen FC (2014). Functional characterization of BRCA1 gene variants by mini-gene splicing assay. Eur J Hum Genet.

[24] Colombo M, Blok MJ, Whiley P, Santamarina M, Gutierrez-Enriquez S, Romero A (2014). Comprehensive annotation of splice junctions supports pervasive alternative splicing at the BRCA1 locus: a report from the ENIGMA consortium. Hum Mol Genet.

